# A transpupillary approach for crosslinking Guinea pig sclera using WST11 and near-infrared light

**DOI:** 10.1038/s41598-026-36438-w

**Published:** 2026-01-24

**Authors:** Demi H. J. Vogels, Yusupjan Abdulla, William Myles, Sara Cummings, Lilach Agemy, Tamar Yechezkel, Arie L. Marcovich, Avigdor Scherz, Vanessa L. S. LaPointe, Sally A. McFadden, Mor M. Dickman

**Affiliations:** 1 Department of Cell Biology–Inspired Tissue Engineering, MERLN Institute for Technology-Inspired Regenerative Medicine, Maastricht, The Netherlands; 2 School of Psychology, The University of Newcastle, Callaghan, NSW Australia; 3 Department of Plant and Environmental Sciences, The Weizmann Institute of Science, Rehovot, Israel; 4 Present Address: Department of Ophthalmology, Kaplan Medical Center, Rehovot, Israel; 5 Faculty of Medicine, Hebrew University of Jerusalem, Jerusalem, Israel; 6 Present Address: Department of Ophthalmology, Utrecht University Medical Center, Utrecht, The Netherlands; 7 Present Address: Ocular Imaging Research Group, Singapore Eye Research Institute, Duke-NUS Medical School, Singapore, Singapore; 8 Present Address: Department of Ophthalmology, Yong Loo Lin School of Medicine, National University of Singapore, Singapore, Singapore

**Keywords:** Drug delivery, Tissues, Drug delivery, Drug safety, Eye diseases, Scleral diseases, Drug development, Biomedical engineering, Tissues, Mechanical properties, Lasers, LEDs and light sources

## Abstract

Crosslinking strengthens the sclera and holds potential as a treatment for myopia. This study aims to identify optimal crosslinking parameters in guinea pigs using WST11 with dextran followed by near-infrared (NIR) illumination. Guinea pig eyes were incubated in WST11 with 2, 5 or 10% dextran, and penetration depth was assessed by fluorescence microscopy. Crosslinking efficacy was measured as thermal stability using a thermal degradation assay, following incubation in WST11 + 10% dextran (WST-D) for 30 min and NIR irradiation at 10 mW/cm^2^ or 20 mW/cm^2^ for 10, 20 and 30 min. The optimized parameters were then applied in vivo in 6-month-old guinea pigs. *Ex vivo* treatment using the optimal crosslinking parameters (WST-D, 30 min; NIR, 10 mW/cm^2^, 30 min) resulted in the highest thermal degradation midpoint (*ΔT*_*50*_: 6.8), significantly higher than untreated controls (*p* = 0.0006), with WST-D penetration limited to the sclera. Efficacy was greater in eyes obtained from older compared to younger guinea pigs (*p* = 0.02). *In vivo*, WST-D/NIR treatment resulted in significant crosslinking compared to untreated controls (equatorial, *ΔT*_*50*_: 3.7, *p* < 0.0001; posterior, *ΔT*_*50*_: 3.4, *p* = 0.01). WST-D/NIR treatment effectively induces scleral crosslinking, with age-related differences suggesting the need for personalized treatment.

**Supplementary Information:**

The online version contains supplementary material available at 10.1038/s41598-026-36438-w.

## Introduction

Progressive myopia remains a significant public health problem due to its increasing prevalence, ocular complications, and status as a leading cause of blindness^1,2^. The excessive axial elongation in progressive myopia is associated with scleral remodeling, resulting in a thinner and biomechanically weaker sclera^3,4^. Despite advances in slowing myopia progression by lenses, pharmaceutical agents, and posterior scleral reinforcement, the benefits are often small and short-lived or come with significant side effects^5^. Moreover, no treatments exist to address the underlying weakening of the scleral collagen fibril matrix or effectively treat pathological myopia. Recent research has focused on scleral collagen crosslinking, which can biomechanically strengthen the weakened sclera, potentially halting myopia progression and treating its pathologies by addressing the underlying cause.

Collagen crosslinking was first introduced by Wollensak et al. using a light-activated approach, involving riboflavin (RF) impregnation followed by ultraviolet A (UVA) irradiation^6^. Light-activated crosslinking has the advantage of limiting the crosslinking to the area of interest. However, invasive surgical procedures are needed to reach the sclera with external UVA light. Furthermore, while crosslinking by RF/UVA (370 nm) at 4.2 mW/cm^2 ^for 30 min significantly increased the stiffness of porcine and human sclera^6^, serious retinal damage was found in rabbit eyes^7^. Reducing the UVA irradiation parameters to (365 nm at 3 mW/cm^2 ^for 40 min) could increase the scleral stiffness without retinal damage^8,9^; however, these parameters were inadequate in the thicker human and porcine sclera^9^. These limitations, including the need for surgery, retinal damage, and low penetration depth of UVA light, which is especially important in treating the thicker posterior sclera, highlight the need for alternative crosslinking methods.

As an alternative to light-activated crosslinking, chemical crosslinking agents, such as glyceraldehyde and genipin, can also increase scleral biomechanical stiffness^10–12^and heat resistance^13^, and slow axial elongation^13–17^without the need for surgery or risk of phototoxicity. However, they suffer from several drawbacks, including their lack of target specificity, as their diffusion can lead to crosslinking in unwanted areas^12,17^. Moreover, chemical crosslinking poses potential risks such as increased intraocular pressure, glaucomatous changes^16^, histological alterations^16,18^, inflammatory responses^10^, fibrosis^12^, retinal toxicity^12,18^, and variable efficacy dependent on dosage and frequency of injections^15,17^. Lastly, all studies using chemical crosslinking required repeated injections of crosslinking agents to effectively slow myopia progression^13–17^.

We recently reported that the bacteriochlorophyll-derived photosensitizer WST11 with dextran (WST-D) activated with near-infrared (NIR) irradiation effectively stiffened rabbit corneas^19^. We hypothesize that WST-D/NIR crosslinking may provide a safe and effective method to strengthen the sclera, potentially overcoming the current challenges associated with scleral crosslinking. By using NIR light at a safe wavelength of 753 nm, light can be directed through the pupil, allowing for deep penetration into the sclera potentially without inducing cytotoxicity and without the need for surgery, making it a noninvasive treatment. Addition of dextran could also limit the penetration depth of the photosensitizer to only the sclera. Notably, systemic WST11 administration received marketing authorization from the European Medicines Agency for vascular-targeted photodynamic therapy of prostate cancer, which makes the treatment promising for clinical translation. This study aimed to identify the parameters needed to achieve optimal efficacy of WST-D/NIR treatment in guinea pig sclera. The guinea pig is a well-established animal model for myopia^20,21^, which has commonly been used in scleral crosslinking studies^21–29^. While myopia was not induced in the current study, establishing effective treatment parameters in guinea pig sclera is valuable for future *in vivo* studies investigating the ability of WST-D/NIR to slow myopia progression. We used fluorescence microscopy to measure the penetration depth of WST-D into the sclera and a thermal degradation assay to analyze treatment efficacy in terms of thermal stability. Thermal stability serves as a validated proxy for collagen crosslinking and correlates with increased enzymatic resistance^30^. Consistent with this, WST11/NIR treatment has previously been shown to increase enzymatic resistance in corneal tissue^31^. We identified optimal WST-D/NIR treatment parameters that resulted in enhanced scleral thermostability, suggesting optimal parameters for scleral crosslinking, which offers a promising foundation for future investigations towards clinical translation.

## Results

### Dextran limited the penetration of WST11 to the sclera

**Fig. 1:**
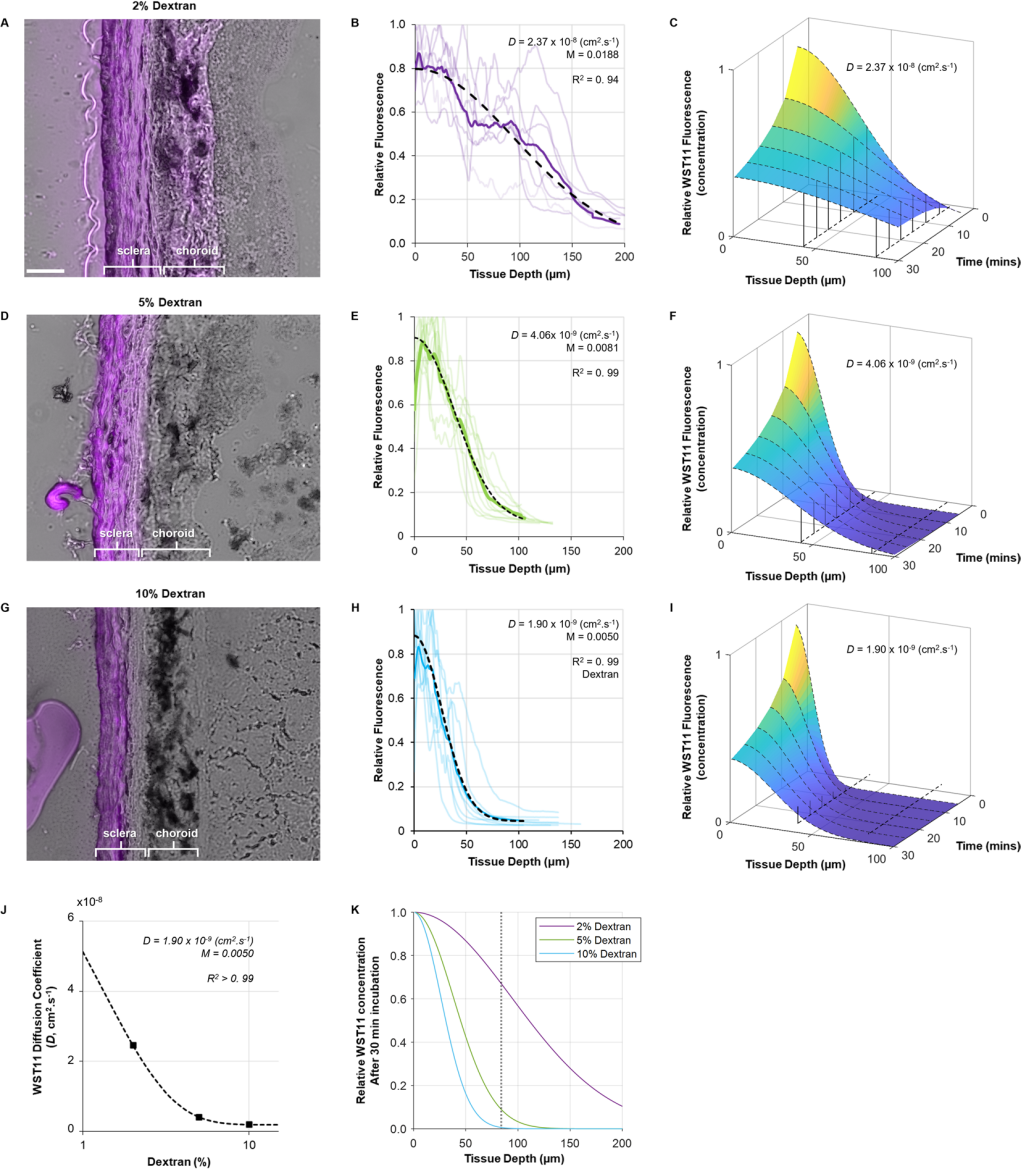
*Ex vivo *penetration depth of WST11 formulated with different concentrations of dextran. (**A**, **D**, **G**) Representative images of scleral–choroid sections of guinea pig eyes posteriorly incubated in WST-D (purple) solutions containing 2%, 5%, or 10% dextran (*N* = 3 eyes per group) for 30 min, showing full penetration of WST-D into the sclera and choroid, complete penetration into the sclera, and penetration halfway through the sclera, respectively. Scale bar: 50 μm. (**B**, **E**, **H**) Quantitative analysis depicting the normalised fluorescence intensity of WST-D measured in the sclera-choroid for solutions containing 2% (**B**, purple), 5% (**E**, green), or 10% (**H**, blue) dextran. Solid lines represent the mean decline in fluorescence intensity from seven posterior measurements (semi-transparent traces) made for each eye. Dashed lines depict fits of the diffusion model of Eq. (1) to the mean fluorescence intensity for each dextran concentration. (**C**, **F**, **I**) Diffusion modelling of WST-D penetration in the guinea pig sclera for formulations containing 2% (**C**), 5% (**F**) or 10% (**I**) dextran, depicting the relative fluorescence intensity (relative concentration) of WST11 (y-axis), per tissue depth (x-axis) with incubation time (z-axis). Dashed lines depict the average thickness of the posterior sclera (80 μm) and peripheral sclera (40 μm) for reference. (**J**) Nonlinear curve fit of the diffusion coefficient (*D*) of WST11 in the sclera as a function of dextran concentration, used to estimate the IC_50_ value. (**K**) Relative WST11 fluorescence per tissue depth for solutions containing 2% (purple), 5% (green) and 10% (blue) dextran following 30 min incubation based on the models depicted in panels C, F, and I, respectively.

To control the penetration of WST11 into the sclera and thereby ensure the treatment solution does not reach other ocular structures, dextran of increasing concentrations was added to the WST11 formulation. Either 2%, 5%, or 10% dextran was added (*N* = 3 per group), and the penetration depth of WST11 (purple) into the eye after 30 min of posterior incubation was assessed by fluorescence microscopy (Fig. 1). Overall, WST11 fluorescence peaked at the superficial scleral stroma, just beneath the episcleral surface for all dextran percentages. As expected, increasing the dextran concentration reduced the penetration depth (and fluorescence) of WST11 in the posterior sclera (Fig. 1A, D, G). This was consistent with a corresponding decrease in the diffusion coefficient of WST11 in the sclera, indicating a reduced molecular mobility at higher dextran concentrations (Fig. 1B, E, H). In the presence of 2% dextran, WST11 completely penetrated the sclera and choroid (Fig. 1A), with a corresponding diffusion coefficient (*D*) of 2.37 × 10⁻⁸ cm²·s⁻¹ (Fig. 1B). The 5% dextran solution allowed WST11 to pass through the full thickness of the sclera with only limited WST11 observed in the choroid (Fig. 1D), consistent with a lower *D* of 4.06 × 10^− 9^ cm²·s⁻¹in the sclera (Fig. 1E). The 10% dextran solution further reduced the *D* of WST11 to 1.90 × 10^− 9^ cm²·s⁻¹, limiting the bulk of WST11 to the outer half of the posterior sclera.

A diffusion depth model was produced for WST11, using the diffusion coefficient measured for each dextran-containing solution, to determine the expected time it would take for the sclera–choroid boundary (80 μm) to contain 1% of the maximum WST11 concentration present in the sclera (Fig. 1C, F, I). With 2% dextran, this time was < 3 min (Fig. 1C). By comparison, this threshold was not reached until 15 min for the 5% dextran solution (Fig. 1F), and not achieved within the first 30 min for the 10% dextran solution (Fig. 1I). Based on these measurements, the IC_50_ for dextran, defined as the concentration that reduces WST11 diffusion in the sclera by 50%, was approximately 1.7% (Fig. 1J). Therefore, the 10% dextran formulation combined with an incubation time of 30 min was chosen for subsequent experiments, to limit the WST11 penetration exclusively to the sclera.

### WST-D/NIR treatment of guinea pig sclera enhances crosslinking efficacy, protecting against thermal degradation, with a greater effect in older animals

**Fig. 2:**
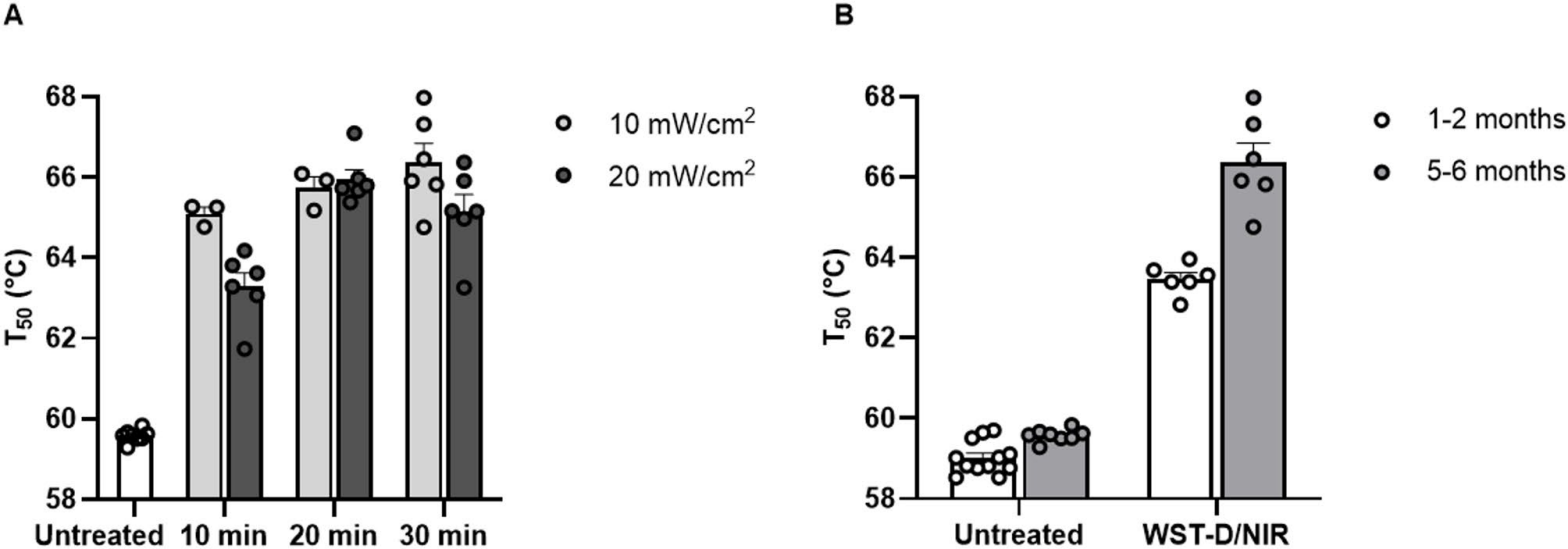
*Ex vivo* efficacy of WST-D/NIR scleral treatment measured by a thermal degradation assay, where a higher temperature required for 50% tissue degradation (*T*_*50*_) indicates greater crosslinking efficacy. (**A**) Eyes from 5- to 6-month-old guinea pigs were treated with WST-D for 30 min *ex vivo*, followed by NIR illumination at 10 mW/cm² for 10 min (*N* = 3), 20 min (*N* = 3) or 30 min (*N* = 6), and at 20 mW/cm² for 10 min (*N* = 6), 20 min (*N* = 6), or 30 min (*N* = 6). All treated sclera showed a significant increase in *T*_*50*_ compared to untreated controls (*p* ≤ 0.01). No significant differences were found comparing the different times across both laser powers, or between the powers when comparing all time points together. However, when comparing all groups, NIR illumination at 10 mW/cm^2^ for 20–30 min and at 20 mW/cm^2^ for 20 min had significantly higher *T*_*50*_ values compared to NIR illumination at 20 mW/cm^2^ for 10 min (*p* ≤ 0.04). (**B**) Sclera from 1- to 2-month-old guinea pigs treated with WST-D for 30 min, followed by NIR illumination at a power of 10 mW/cm^2^ for 30 min, showed a significant increase in *T*_*50*_ compared to untreated sclera (*p* < 0.0001). But when compared to the sclera from the older 5- to 6-month old guinea pigs, the effect was significantly lower (*p* = 0.02). Each data point represents an individual eye. Sigmoidal curves used to derive the *T*_*50*_ values for untreated controls (Supplementary Fig. 1) and for all treatment conditions (Supplementary Fig. 2), as well as comparisons of fitted sigmoidal curves for all treatment conditions (Supplementary Fig. 7 A, B), are provided in the supplementary material.

To optimize the NIR irradiation power and time, eyes from 5- to 6-month-old guinea pigs were incubated in WST-D for 30 min, followed by scleral NIR illumination at 10 mW/cm² for 10 min (*N* = 3), 20 min (*N* = 3), 30 min (*N* = 6), or 60 min (*N* = 6), and at 20 mW/cm² for 10 min (*N* = 6), 20 min (*N* = 6), or 30 min (*N* = 6) (Fig. 2A; Table 1). For the NIR illumination at 10 mW/cm^2^, an extended illumination time of 60 min was included to assess if the efficacy of the treatment would reach a plateau at prolonged exposure. To compare the efficacy of the various NIR illumination settings, a thermal degradation assay was used. This assay evaluates collagen crosslinking by measuring tissue resistance to heat-induced shrinkage. When collagen fibers are heated, they begin to denature and contract; crosslinked collagen is more thermally stable and therefore shrinks at a higher temperature. To assess treatment efficacy, we determined the temperature required for 50% tissue shrinkage (*T*_*50*_), with a higher *T*_*50*_ indicating greater crosslinking. Hereby, *ΔT*_*50*_ is the change in *T*_*50*_ as compared to untreated control. All WST-D–treated sclera, followed by various irradiation times and NIR powers, had significantly increased *T*_*50*_ compared to untreated controls: at 10 min (10 mW/cm^2^, *ΔT*_*50*_: 5.5, *p* = 0.006; 20 mW/cm^2^, Δ*T*_*50*_: 3.7, *p* = 0.003), 20 min (10 mW/cm^2^, *ΔT*_*50*_: 6.2, *p* = 0.01; 20 mW/cm^2^, *ΔT*_*50*_: 6.4, *p* < 0.0001), 30 min (10 mW/cm^2^, *ΔT*_*50*_: 6.8, *p* = 0.0006; 20 mW/cm^2^, *ΔT*_*50*_: 5.6, *p* = 0.001), and 60 min (10 mW/cm^2^, *ΔT*_*50*_: 6.9, *p* = 0.001). No significant differences were found comparing the different times across both laser powers, or between the powers when comparing all time points together. However, when comparing all groups, several NIR illumination conditions significantly increased the *T*_*50*_ value compared to NIR illumination at 20 mW/cm^2^ for 10 min: at 20 min (10 mW/cm^2^, *p* = 0.02; 20 mW/cm^2^, *p* = 0.005), 30 min (10 mW/cm^2^, *p* = 0.02), and 60 min (10 mW/cm^2^, *p* = 0.04). These data show that longer incubation at lower power was superior to short incubation at higher power. Also, NIR illumination at 10 mW/cm^2^ for 30 min yielded the highest *T*_*50*_ value, indicating target parameters for treatment. Sigmoidal curves used to derive the *T*_*50*_ values for untreated controls (Supplementary Fig. 1) and all treatment conditions (Supplementary Fig. 2A–G) and comparisons of the fitted sigmoidal curves across all treatment conditions are presented in the supplementary information (Supplementary Fig. 7 A).

**Table 1:** Overview of experimental conditions, including treatment groups, animal age and sample size (N) for each experiment. For relevant experiments, the dextran percentage, incubation time, laser power, laser illumination time and treatment location were specified. The untreated groups in the thermal degradation assay serve as references for the same-age groups in this assay (experiments 2.1–2.4).

1.* Ex vivo* penetration depth			
Dextran (%)		Age (months)	*N* (animals)
2		1	3
5		1	3
10		1	3

2. Thermal degradation assay			
		Age (months)	*N* (animals)
Untreated		1–2	6
		5–6	8

2.1 *Ex vivo* laser optimization			
Power (mW/cm2)	Illumination time (min)	Age (months)	*N* (animals)
10	10	5–6	3
	20	5–6	3
	30	1–2	6
	30	5–6	6
	60	5–6	6
20	10	5–6	6
	20	5–6	6
	30	5–6	6

2.2 *In vivo* clearance of WST11			
Treatment	Incubation time (h)	Age (months)	*N* (animals)
Saline/NIR	0.5	5	2
	24	5	1
WST/NIR	0.5	5	4
	5	5	1
	24	5	1
	24 post mortem	5	1

2.3* Ex vivo* transpupillary illumination			
Treatment		Age (months)	*N* (animals)
Saline/NIR		1–2	5
WST-D/NIR		1–2	6

2.4* In vivo* efficacy			
Treatment	Location	Age (months)	*N* (animals)
Saline-D/NIR	temporal equatorial	6	6
Contralateral control	nasal equatorial		
WST-D/NIR	temporal equatorial	6	6
Contralateral control	nasal equatorial		
WST-D/NIR	temporal posterior	6	6
Contralateral control	nasal posterior		

3. Tensile testing			
Treatment		Age (months)	*N* (animals)
Untreated		1–2	8
WST-D		1–2	8
Saline/NIR		1–2	8
WST-D/NIR		1–2	8

To confirm the efficacy of the treatment in sclera from younger 1- to 2-month-old guinea pigs, we incubated eyes for 30 min in WST + 10% dextran followed by NIR illumination of the sclera at a power of 10 mW/cm^2^ for 30 min. This resulted in a significant increase in *T*_*50*_ compared to untreated sclera (*ΔT*_*50*_: 4.5, *p* < 0.0001) (Fig. 2B). The corresponding sigmoidal curves used to derive the *T*_*50*_ values for the younger guinea pigs are provided in the supplementary information (Supplementary Fig. 2H). When compared to sclera from 5-month-old guinea pigs treated with the same conditions, the effect was significantly lower (*p* = 0.02). Comparisons of the fitted sigmoidal curves for the effect of age on treatment efficacy are presented in the supplementary information (Supplementary Fig. 7B).

To further validate the crosslinking efficacy of WST-D/NIR treatment, *ex vivo* biomechanical testing was performed on sclera from 1- to 2-month-old guinea pig eyes (*N* = 8 per group). The treatment groups were: untreated, WST11 + 10% dextran incubation for 30 min (WST-D), saline incubation followed by NIR illumination at 10 mW/cm² for 30 min (saline/NIR), and WST11 + 10% dextran incubation for 30 min followed by NIR illumination at 10 mW/cm² for 30 min (WST-D/NIR). Pairwise comparisons showed no significant differences between untreated, saline/NIR, and WST-D groups (*p* > 0.05), indicating that NIR illumination or WST-D incubation alone did not alter scleral stiffness. In contrast, WST-D/NIR treatment resulted in a significantly higher Young’s modulus compared to untreated controls (*p* = 0.001), saline + NIR (*p* = 0.007), and WST-D alone (*p* = 0.007) (Supplementary Fig. 3). These biomechanical findings are consistent with the thermal degradation results, confirming that the thermal assay reliably reflects changes in tissue stiffness.

### Time-dependent retention of WST11 in the guinea pig sclera

This experiment aimed to assess the* in vivo* retention of WST11 in the guinea pig sclera over time and to determine whether its disappearance was due to passive diffusion or active clearance. WST11 was applied for either 30 min, 5 h, or 24 h, after which the animals were euthanized, the eyes immediately enucleated, and NIR illumination was performed. Thermal degradation analysis revealed an increase in *T*_*50*_ with 30-min of WST11 incubation compared to untreated controls (Δ*T*_*50*_: 4.28; *N* = 4 eyes). No significant change in *T*_*50*_ was observed after 5-h and 24-h incubation of WST11, with only a minimal increase at 5 h (*ΔT*_*50*_: 1.22; *N* = 1 eye) and 24 h (*ΔT*_*50*_: 0.65; *N* = 1 eye). These changes were similar to those observed in the saline/NIR controls at 30 min (*ΔT*_*50*_: 0.74; *N* = 2 eyes) and 24 h (*ΔT*_*50*_: 0.11; *N* = 1 eye).

To investigate whether WST11 loss could be attributed to passive diffusion or active clearance, an additional post-mortem experiment was performed. In this setup, WST11 was applied* in vivo* for 30 min, but instead of immediate enucleation after euthanasia, the animal was euthanized and the eyes remained in situ for 24 h before enucleation and NIR irradiation. The sclera showed a similar increase in *T*_*50*_ as the 30 min group with immediate NIR irradiation (*ΔT*_*50*_: 3.89; *N* = 1 eye). Sigmoidal curves used to derive the *T*_*50*_ values (Supplementary Fig. 4) and comparisons of fitted sigmoidal curves for all treatment conditions are provided in the supplementary information (Supplementary Fig. 7 C).

In addition to the thermal data, the sclera in the 30-minute and 24-hour post-mortem groups appeared opaque rather than translucent, consistent with WST11 retention, whereas the 5-hour* in vivo* group appeared only slightly opaque, and the 24-hour *in vivo* group appeared fully translucent, both resembling the saline and untreated controls. The observed opacity reflects the presence of WST11, which absorbs in the near-infrared range and therefore reduces scleral translucency. The observed opacity reflects the presence of WST11, which absorbs in the near-infrared range and therefore reduces scleral translucency. Representative images illustrating these findings are provided in the Supplementary Information (Supplementary Fig. 8).

### *In vivo* WST-D/NIR treatment shows high efficacy in both equatorial and posterior guinea pig sclera

Before conducting the *in vivo* study, we first validated the efficacy of transpupillary illumination *ex vivo* using whole enucleated eyes from 1- to 2-month-old guinea pigs. Eyes were incubated for 30 min in either WST-D (*N* = 6) or saline (*N* = 5) followed by transpupillary NIR illumination at 10 mW/cm^2^ for 30 min, targeting the equatorial sclera. The *ex vivo* transpupillary WST-D/NIR treatment significantly increased *T*_*50*_ compared to both the untreated controls (*ΔT*_*50*_: 5.3, *p* < 0.0001) and the saline/NIR controls (*p* < 0.0001). No significant difference was observed between saline/NIR controls and untreated controls. Additionally, no significant differences were found when comparing *ex vivo* transpupillary NIR light delivery to direct NIR light delivery. Sigmoidal curves used to derive the *T*_*50*_ values (Supplementary Fig. 5) and comparisons of fitted sigmoidal curves for all treatment conditions are provided in the supplementary information (Supplementary Fig. 7D). Although younger animals were used here due to availability, these experiments were valuable for confirming the feasibility and efficacy of transpupillary delivery, as the significant increase in *T*_*50*_ observed in 1–2-month-old animals indicated that this method was effective and likely to yield even stronger results in older animals. To further support the feasibility of transpupillary delivery, we estimated NIR light transmission through the guinea pig sclera using the Beer–Lambert law. This analysis showed that approximately 40% of the NIR light remains at the outer scleral surface for a posterior scleral thickness of ~ 80 μm, supporting sufficient light availability for crosslinking. This estimate is likely conservative, as it assumes diffuse light propagation; in contrast, our system uses focused NIR light, which enhances localized penetration and reduces scattering losses.

**Fig. 3:**
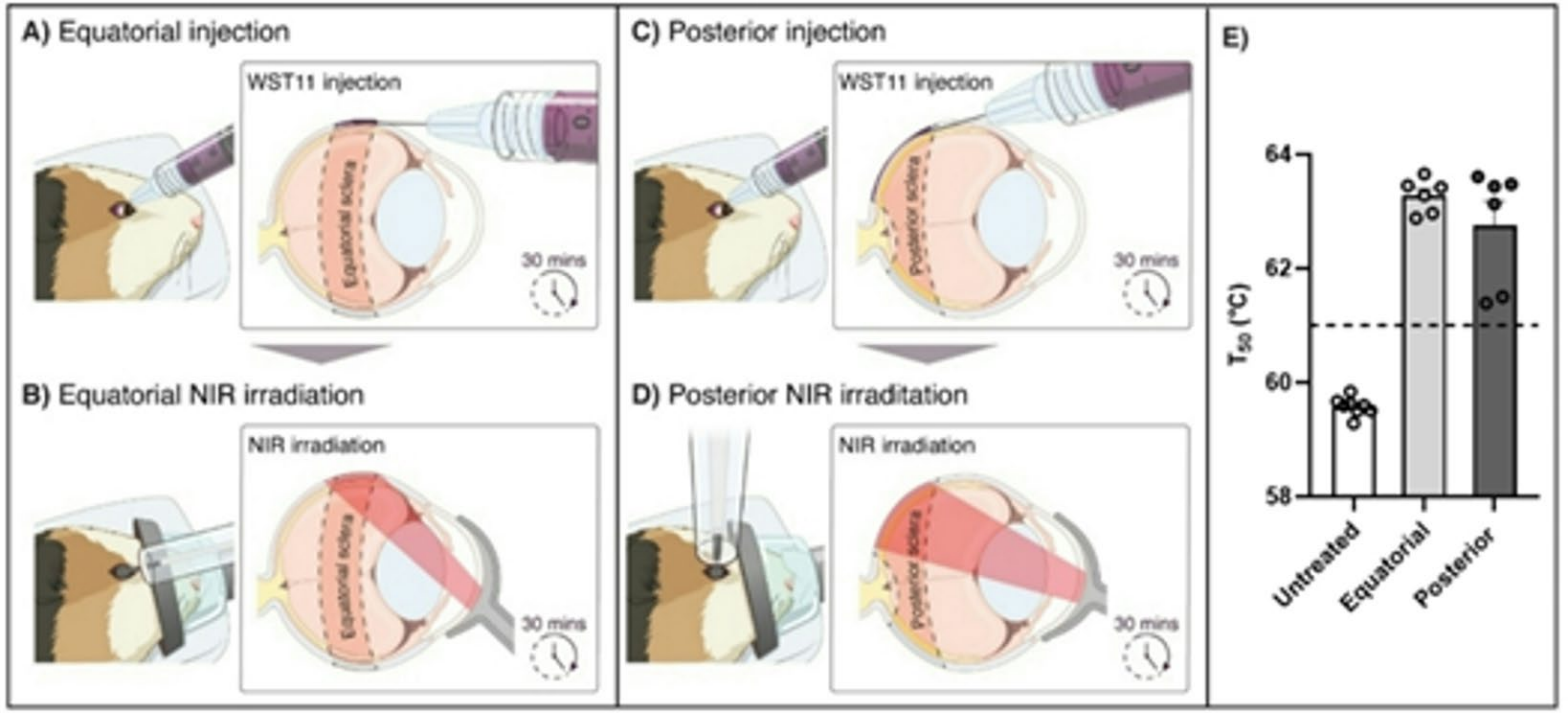
The* in vivo* efficacy of WST-D/NIR treatment on the equatorial and posterior sclera was investigated in 6-month-old guinea pigs. (**A**,** C**) Schematic of the sub-Tenon injection of WST-D delivered to the equatorial and posterior sclera, respectively. (**B**, **D**) Thirty minutes after injection, NIR transpupillary irradiation at 15 mW/cm^2^ for 30 min was performed at the injection site on the equatorial or posterior sclera, respectively. (**E**) Both equatorial (*N* = 6) and posterior (*N* = 6) WST-D/NIR treatment resulted in a significant increase in *T*_*50*_ compared to untreated controls (*N* = 8; *p* ≤ 0.01). No difference was observed comparing the treated locations. When compared to saline-D/NIR controls (*N* = 6), represented by the dashed line, only the equatorial treatment group showed a significant increase in *T*_*50*_ (*p* < 0.001). For the saline-injected controls, guinea pigs received a sub-Tenon injection of saline + 10% dextran followed by NIR transpupillary irradiation at 15 mW/cm^2^ for 30 min performed on the equatorial sclera. Each data point represents an individual eye. Sigmoidal curves used to derive the *T*_*50*_ values (Supplementary Fig. 6) and comparisons of fitted sigmoidal curves for all treatment conditions are provided in the supplementary material (Supplementary Fig. 7E).

To determine the efficacy of the WST-D/NIR treatment* in vivo*, WST-D was first administered via sub-Tenon injection targeting the equatorial sclera (Fig. 3A), followed by transpupillary NIR irradiation at 15 mW/cm^2^ for 30 min directed at the equatorial sclera (Fig. 3B). In a second group, WST-D was delivered by retrobulbar injection to target the posterior sclera (Fig. 3C), followed by transpupillary NIR irradiation of the posterior sclera under identical conditions (Fig. 3D), in 6-month-old guinea pigs (*N* = 6 per group). While aiming for the optimal NIR power of 10 mW/cm^2^, as identified *ex vivo*, the estimated power at the scleral level during *in vivo* transpupillary illumination was calculated to be 15 mW/cm^2^. Both equatorial and posterior scleral treatment by WST-D/NIR significantly increased *T*_*50*_ compared to untreated controls (equatorial, *ΔT*_*50*_: 3.7, *p* < 0.0001; posterior, *ΔT*_*50*_: 3.2, *p =* 0.01) (Fig. 3E). No significant difference was observed between treated equatorial and posterior sclera. Interestingly,* in vivo* equatorial saline-D/NIR controls (*N* = 6) had significantly increased *T*_*50*_ when compared to untreated controls (*ΔT*_*50*_: 1.4, *p* = 0.002). When comparing the WST-D/NIR treatment to saline-D/NIR controls, the efficacy was still significantly higher for the equatorial treatment (*p* < 0.0001), whereas no significant difference was observed for the posterior treatment (*p* = 0.09). Sigmoidal curves used to derive the *T*_*50*_ values (Supplementary Fig. 6A–C) and comparisons of fitted sigmoidal curves for all treatment conditions are provided in the supplementary information (Supplementary Fig. 7E).

To assess the localized or potential general effect beyond the treatment area, scleral punches from the contralateral nasal side of the temporal WST-D/NIR treatment and saline-D/NIR control were also analyzed. The equatorial treatment resulted in significantly higher crosslinking efficacy compared to the contralateral untreated side (*p* = 0.0002). The posterior treatment was similar between the treated side and the contralateral untreated side. For the saline-D/NIR control group, the contralateral untreated side was similar to the treated side. When comparing the contralateral controls to the untreated controls, a significant increase in *T*_*50*_ was observed for the contralateral equatorial (*ΔT*_*50*_: 1.5, *p* = 0.005) and contralateral saline-D/NIR control (*ΔT*_*50*_: 1.3, *p* = 0.008), whereas no difference was observed for the contralateral posterior control (*ΔT*_*50*_: 1.6, *p* = 0.08). Sigmoidal curves used to derive the *T*_*50*_ values are provided in the supplementary material (Supplementary Fig. 6D–F). These data indicate that WST-D/NIR treatment was localized with equatorial treatment but not with posterior treatment. Also, saline-D/NIR control treatment increased the protection against thermal degradation compared to untreated controls.

## Discussion

This study demonstrates the potential of WST-D/NIR in the treatment of myopia by enhancing scleral thermal stability as a measure of crosslinking. While previous crosslinking studies have shown several drawbacks, including phototoxicity^7^, the need for invasive surgical exposure of the sclera^7,9^, low light penetration^9^, and lack of scleral specificity^12,17^, our study indicates that WST-D/NIR treatment of the sclera overcomes these challenges. We showed that WST-D/NIR treatment of guinea pig sclera* in vivo* significantly increased scleral thermal stability, an indirect measure of crosslinking. We used a deep-penetrating wavelength (753 nm), which enables illumination of the sclera through the pupil, eliminating the need for invasive procedures and potentially reducing patient recovery time and the risk of complications. Additionally, dextran modulated the penetration depth of WST11, with 10% dextran effectively localizing treatment to the sclera, reducing potential off-target effects. This observation is consistent with the diffusion-limiting properties of dextran, which increases viscosity and acts as a molecular sieve^32,33^, restricting the diffusion of WST11 molecules through the tissue. This observation is in line with our previous study in rabbits, where 20% dextran limited the penetration of WST11 to half of the sclera^34^. The adaptability of penetration depth is especially beneficial for accommodating variations in scleral thickness between patients, especially in high myopia, where the sclera is thinner.

The guinea pig was selected as a preclinical model due to its well-established role in myopia research and its relevant ocular and scleral features. Guinea pigs reliably develop axial elongation and refractive shifts in response to form deprivation^20^, showing changes that resemble human pathologic myopia, including axial elongation, scleral thinning, disorganization of collagen lamellae^35^, and regional biomechanical changes^36^. While scleral thickness increases from guinea pig^37^to rabbit^38^, to porcine^39^, and is greatest in humans^40^, the use of dextran allowed controlled localization of treatment, enhancing the translational value of this model. Furthermore, guinea pig eyes undergo spontaneous myopia progression, with remodeling patterns similar to early-stage pathologic myopia in children^41^, and demonstrate axial growth, emmetropization^42^, and retinal development^43^ comparable to humans. These similarities support the use of guinea pigs as a preclinical model to optimize scleral crosslinking for the treatment of myopia.

In terms of safety, we recognize the novelty of transpupillary NIR delivery and the importance of addressing potential risks, particularly to the retina. Although this study did not include direct retinal or choroidal imaging, electroretinogram (ERG), or histology, several independent safety considerations support the premise that these structures are not at risk under the parameters used. First, the applied NIR exposure is well below the International Commission on Non-Ionizing Radiation Protection (ICNIRP) ocular safety limits for 753 nm light and far lower than exposure levels previously shown to be safe *in vivo*^44^. In prior studies using intravenous WST-11, illumination of the retina with light power density more than an order of magnitude higher than that used here did not produce retinal structural damage at doses ≤ 2.5 mg/kg^45^. Importantly, in contrast to intravenous delivery, the WST-D formulation in our study is applied externally on the sclera, and our fluorescence imaging demonstrates that the drug does not penetrate beyond the scleral wall. This is consistent with prior corneal work showing that extraocular application results in systemic concentrations orders of magnitude below those required for any photochemical or photothermal effect. Together, these findings indicate that no meaningful drug–light overlap occurs at the retina, and therefore no retinal toxicity is expected under the illumination parameters used. We nonetheless emphasize that future *in vivo *work should include direct structural and functional retinal assessments.

Further supporting the safety of this approach, our preliminary observations suggest that WST11 retention in the guinea pig sclera is time-dependent and short-lived* in vivo*, with no detectable crosslinking when NIR irradiation was applied 5–24 h after administration. When eyes remained in situ for 24 h post-mortem, crosslinking efficacy remained similar to the 30-min incubation group with immediate NIR irradiation, suggesting active clearance rather than passive diffusion. Although these findings are based on a small number of eyes and should be interpreted with caution, they support the idea that biological clearance may prevent prolonged exposure with potential side effects. Together, these factors provide a solid foundation for the safety of transpupillary WST-D/NIR treatment in the sclera. Nevertheless, future studies should include direct evaluation of retinal safety using imaging modalities such as OCT and ERG, as well as histopathological analysis, to confirm its safety profile.

Both *ex vivo* and* in vivo* WST-D/NIR treatments were efficacious in enhancing the thermostability of guinea pig sclera. The *in vivo* efficacy was observed for both equatorial and posterior sclera, indicating the versatility of the treatment. In line with our results, two other studies demonstrated that RF/UVA crosslinking is effective in both equatorial and posterior sclera^46,47^. However, while our study used thermostability as a measure of regional differences in efficacy, the other studies used Young’s modulus and reported contrasting results. One study found that the Young’s modulus was relatively higher in the equatorial compared to posterior sclera, likely due to the greater thickness of the latter^46^. As UVA illumination has a limited penetration depth^9^, a larger proportion of the equatorial sclera was treated compared to the posterior sclera, resulting in a greater increase in relative stiffness. However, the opposite result was found in another study, where the posterior sclera showed a higher relative effect compared to the equatorial sclera^47^, which contradicts expectations. Notably, the reported Young’s modulus values for human sclera were unusually high (200 to 470 MPa) values more typical of tendon (330 to 822 MPa)^48,49^, raising concerns about the reliability of those measurements. As NIR light allows for deep tissue penetration^50–52^, our results suggest that our treatment mitigates variability in proportional treatment outcomes.

The equatorial treatment produced a pronounced localized effect in the treated temporal region. Interestingly, the contralateral untreated nasal region also showed a mild increase in stability, significantly higher than in completely untreated eyes (from separate animals) and comparable to NIR-only controls, suggesting that NIR light may induce a mild, uniform stiffening effect across the sclera. The effect of NIR light on scleral stiffening has not been investigated before, but, a meta-analysis showed that repeated, low-level, red-light therapy (RLRL) by either red or NIR light may effectively delay myopia progression, although the certainty of this evidence was low and a rebound effect may occur^53^. RLRL has been shown to activate the Smad pathway via increased ATP and TGF-β expression^54^, which may promote scleral remodeling via the overproduction of collagen I alpha1 to mediate myopia progression^55^. The suggested scleral remodeling induced by NIR light could therefore be the mechanism behind the general stiffening effect that we observed in our treatment.

Our study also revealed age-related differences in the thermostability following WST-D/NIR treatment, with sclera from 5-month-old guinea pigs showing greater protection against thermal degradation compared to sclera from 1-month-old guinea pigs. One possible explanation is that the extracellular matrix (ECM) in the 5–6-month-old group, roughly equivalent to young adults aged ~ 20–25 years in humans based on guinea pig sexual maturity at 2.5 months^56^, has reached a more optimal stage of structural and biochemical maturation to support crosslink formation. In contrast, the 1–2-month-old animals, corresponding to ~ 5–8-year-old children, likely have incompletely developed eyes, which may limit treatment efficacy. This is supported by developmental literature showing that the human eye reaches near-adult size only around age 10^57^, and by evidence that scleral ECM components like biglycan and decorin increase steadily from childhood through the third decade before declining after the fourth^58^. Similarly, in the cornea, the greatest increase in elastic modulus following crosslinking was observed in the youngest adult group (26–41 years), compared to older adults^59^, consistent with our findings in the guinea pig sclera. These findings underscore the importance of considering tissue maturity when optimizing crosslinking parameters and suggest that age-specific treatment protocols could improve therapeutic outcomes. Further research is needed to examine the impact of age on scleral crosslinking and whether personalized treatment should be applied to different age groups.

When compared to other existing scleral crosslinking methods, our treatment demonstrated superior efficacy at similar concentrations. In a study comparing multiple crosslinking agents at a concentration of 1 mM, a concentration below their cytotoxic level, including short chain aliphatic β-nitro alcohols (2-nitroethanol, 2-nitro-1-propanol, and 3-nitro-2-pentanol), glutaraldehyde, formaldehyde, and RF/UVA, a shift in *T*_*50*_ of −0.9°C, 0.3 °C, 0.5 °C, 5.0 °C, 2.0 °C and 1.9 °C, respectively, was observed in porcine sclera^60^. The reported shifts in *T*_*50*_ are all lower than the observed shift of 6.8 °C in *T*_*50*_ in our study following WST-D/NIR treatment. A higher concentration of 10 mM of nitro-diols 2-methyl-2-nitro-1,3-propanediol (MNPD) and 2-hydroxymethyl-2-nitro-1,3-propanediol (HNPD) induced higher shifts in *T*_*50*_of 7.9 °C and 10.4 °C, respectively^61^, but raise safety concerns and still lack target specificity as chemical crosslinkers. Another study assessed two photoactivated crosslinking techniques, with Rose Bengal activated by green light inducing a greater *T*_*50*_ shift of 3.3 °C compared to the 1.7 °C shift in *T*_*50*_by RF/UVA in guinea pig sclera^62^, both of which were lower than the shifts observed in the current study. The relatively high increase in *T*_*50*_ of guinea pig sclera following WST-D/NIR treatment in our study suggests a robust enhancement of tissue stability, indicating that our treatment effectively strengthens the sclera.

Despite these promising findings, several limitations must be acknowledged. First, our results in guinea pig sclera may not fully translate to the thicker human sclera. Although thinner than the human sclera, the guinea pig sclera resembles the thinning seen in myopic eyes, supporting its translational relevance. The International Myopia Institute recognizes the guinea pig as a well-established myopia model^63^, though differences in thickness and biomechanics may still limit direct translation. Therefore, more research is needed to assess the efficacy of our treatment in the human eye. Second, we did not use a myopia model, so further research into WST-D/NIR treatment of myopic sclera is needed to assess the treatment’s ability to halt or reverse myopia progression. Nevertheless, the guinea pigs used here provide a foundation for comparison when repeating these studies in a myopic model. Third, while the thermal degradation assay provided an effective measure of crosslinking efficacy, it does not directly quantify mechanical strength or enzymatic resistance. As biomechanical testing was only performed *ex vivo*, future *in vivo* studies should include both mechanical and enzymatic assessments to confirm the treatment’s ability to prevent collagen degradation and axial elongation. Finally, we did not assess safety outcomes such as retinal health following transpupillary WST-D/NIR treatment of the sclera. As this study focused on short-term treatment feasibility, animals were euthanized 30 min after illumination; however, future studies should incorporate longer follow-up to evaluate durability and safety of the treatment. Therefore, future research should address the safety of WST-D/NIR treatment prior to clinical studies. Nevertheless, the current study provides a proof-of-concept and valuable insights into the parameters needed to strengthen the sclera, which form a strong foundation for future studies that can prepare for clinical translation.

In conclusion, our study demonstrated that WST-D/NIR treatment effectively induces scleral crosslinking, which is intended to strengthen the scleral tissue, as evidenced by enhanced thermostability in both the equatorial and posterior sclera. The ability to adjust the penetration depth of WST11 by using dextran and the non-invasive transpupillary NIR irradiation are key advantages over existing scleral crosslinking methods, mitigating the risk of unintended crosslinking of adjacent tissues and phototoxicity. Additionally, our results suggest that age-related differences may play a role in the efficacy of WST-D/NIR treatment, with sclera from older guinea pigs showing an increased thermostability compared to sclera from younger guinea pigs, highlighting the need for personalized treatment.

## Methods

### Animals

All animal procedures were performed according to the Association for Research in Vision and Ophthalmology Statement for the Use of Animals in Ophthalmic and Visual Research^64^. All procedures were approved through the Animal Care and Ethics Committee at The University of Newcastle (Protocol A-2021-136 Cross-linking Treatments for Myopia, Appendix 3). The study also adhered to the ARRIVE guidelines. Tri-colored, short-haired guinea pigs (*Cavia porcellus*) were used for both *ex vivo* and *in vivo* experiments in this study (Table 1). For the *ex vivo* experiments, eyes were obtained from guinea pigs at the research support unit at the John Hunter Hospital (Newcastle, Australia) which were euthanized as part of an unrelated research project. The animals were euthanized using carbon dioxide gas introduced into the euthanasia chamber at a flow rate of 30–70% of the chamber volume per minute. Whole eyes were then enucleated within 10 min of death and transported on dry ice to the University of Newcastle (Newcastle, Australia), where they were stored at −80 °C for further processing. Guinea pigs used for the *in vivo *experiments were bred by the University of Newcastle. The animals were housed with their littermates in plastic boxes with open wire tops on a 12-hour light/dark cycle with free access to food and water. *In vivo *treatments were performed with animals under general anesthesia with either isoflurane (Covetrus Isothesia NXT, ProVet, Melbourne, Australia) in oxygen or by an intraperitoneal injection of 40 mg/kg ketamine hydrochloride (RANDLAB, ProVet) mixed with 5 mg/kg of xylazine hydrochloride (RANDLAB, ProVet).

### WST-D solution

WST11 (Steba Laboratories Ltd., Rehovot, Israel) solution was prepared daily at a concentration of 2.5 mg/ml in saline containing dextran (500 kDa, Pharmacosmos AS, Holbaek, Denmark), and corrected to a pH of 7.2–7.3, after which it was stored in the dark until use. Different concentrations of dextran were prepared by diluting in saline. Solution stability was assessed by measuring the peak wavelength absorbance of the solution between 300 and 900 nm using a spectrometer (Shimadzu UV1900i).

### Imaging WST11 penetration

Eighteen frozen guinea pig eyes, obtained from nine animals, were thawed and incubated with their posterior segment immersed in WST-D solutions (referred to as ‘posterior incubation’), ensuring that diffusion occurred from the outer scleral surface inward. All samples were processed under identical conditions, and comparisons were made exclusively within this consistent experimental framework. Additionally, comparing fresh and frozen human eyes, we observed no noticeable difference in dye diffusion, indicating that freezing at − 80 °C does not substantially alter the permeability characteristics of scleral tissue in this context. Eyes were placed upright in self-manufactured wells with a height corresponding to the average distance from the posterior pole to just below the limbus. This ensured that only the sclera and the underlying choroid and retina were submerged, while the anterior part of the eye remained above the fluid level. This setup provided consistent submersion depth across samples and ensured that WST-D exposure occurred exclusively from the outer surface inward, without potential anterior entry. The eyes were randomly assigned to treatment conditions varying in dextran concentration (2%, 5% or 10%), and incubation time ranged from 5 min to 3 h to assess the diffusion kinetics of WST11 in the sclera. For the 30 min incubation time, each of the three dextran concentrations (2%, 5%, and 10%) was tested with *N* = 3 eyes per condition (Table 1). For the other incubation times (5 min, 15 min, and 3 h), only 10% dextran was used, also with *N*= 3 eyes per condition. As the aim was to assess WST11 penetration depth based on its native fluorescence, no NIR illumination, and therefore no crosslinking, was performed, as photoactivation would quench the dye’s fluorescence signal. After incubation, the eyes were first snap-frozen whole in liquid nitrogen to halt dye diffusion and prevent dye seepage out of the sclera into the embedding media. The frozen eyes were then embedded in chilled optimal cutting temperature compound, which froze on contact with the snap-frozen eyes. Ten-micron–thick sections were cut from the embedded frozen eye using a cryostat at − 20 °C (NX50; Crystar). Sections were cut perpendicular to the surface of the eye in the nasal/temporal plane containing the optic nerve, allowing for imaging of the full thickness of the sclera and choroid. Sections were thaw-mounted onto superfrost plus–coated slides (J1800AMNZ, Ephedia). Images were immediately collected from unmounted frozen sections, to prevent the dye dissolving into the mounting media, using a scanning laser confocal microscope (LSM 900 with Airyscan 2, ZEISS). Composite images used for analysis were created from 10 slices selected from the z-stack using the sum-slices z-project function in ImageJ (version 1.54d, National Institutes of Health, Bethesda, USA). Images were taken from the nasal limbus through the posterior pole and around to the temporal limbus. The relative concentration of WST11 in the sclera was approximated based on the relative fluorescent signal in the CY5 channel^65^, measured perpendicular to the sclera using the plot-profile function in ImageJ. The net diffusion of WST11 through the posterior sclera was modelled as the diffusion of a substance along one axis (in the direction perpendicular to the sclera) following Fick’s second law of diffusion^66,67^. As such, the diffusion coefficient (*D*, cm^2^·s^–1^) of WST11 through the sclera was approximated for each concentration of dextran by fitting the average normalized fluorescent signal through the sclera with the Eq. (1) in MATLAB (R2023a, version 9.14.0.2239454, Update 1; MathWorks, Natick, MA, USA) [y]:

1 $$\:C\left(x,t\right)=\frac{M}{2\sqrt{\pi\:Dt}}\:{exp}^{\left(\frac{-x2}{4Dt}\right)}$$

where *C* is concentration (normalised fluorescence) of WST11, *t* is incubation time (s), and *x* is tissue depth (cm).

The diffusion of WST11 in the sclera and choroid was modelled using the values of *D* measured for each dextran-containing solution by solving Eq. (1) for *t* = 1–30 min, for tissue depth *x* = 1:120 μm. An approximate incubation time required for the sclera-choroid boundary to contain 1% of the maximum WST11 concentration in the sclera was then determined.

The effect of dextran concentration on *D* of WST11 ($\:{D}_{WST11}$) in the sclera was assessed by fitting the results from 30 min incubations with a inhibitory response curve:

2 $$\:{D}_{WST11}={D}_{min}+\frac{{D}_{max}-\:{D}_{min}}{1+{\left(\frac{{C}_{dextran}}{I{C}_{50}}\right)}^{-h}}$$

Where *D*_max_ and *D*_min_ are the respective maximum and minimum diffusion coefficients of WST11 in the sclera, C_dextran_ is the concentration of dextran in solution, *h* is the Hill coefficient and IC_50_ is the concentration at which 50% of the maximum response is observed.

### Light source

A custom-built diode laser (INTERmedic MULTIDIODE 753; Steba Laboratories Ltd., Rehovot, Israel) with a tunable output with a maximum of 1 W at 753 nm was used. For *ex vivo* illumination, NIR light from the optic fiber was applied either directly to a scleral punch or to an enucleated whole eye via transpupillary illumination, achieved using a custom-made transpupillary adapter attached to the optic fiber. The end of the transpupillary adapter consisted of an opaque contact lens which was placed on the cornea of the enucleated guinea pig eye. The same adapter was used for* in vivo* transpupillary illumination, delivering NIR light through the cornea of the treated guinea pig. To estimate the NIR power reaching the sclera during transpupillary illumination, light intensity was measured in fresh enucleated eyes after passing through a pipe lens using a power meter (1918R, Newport Corporation, Irvine, USA). Using different aperture sizes, the adjusted power was calculated based on the distribution of light emerging through the posterior sclera. The power corresponding to the aperture matching the scleral punch size was later used as an estimate for *in vivo* treatment.

### *Ex vivo* WST-D/NIR treatment

Frozen guinea pig eyes were thawed at room temperature and posteriorly immersed in WST-D solution. Both eyes from each animal were used and randomly assigned to different treatment conditions to maximize biological replicates and reduce inter-animal variability (see Table 1 for the number of biological replicates per condition). To validate the use of frozen tissue, we performed baseline experiments comparing fresh and frozen eyes and found no substantial differences in thermal degradation behavior, supporting the reliability of frozen tissue for the thermal degradation assay. After 30 min of incubation, the eyes were dissected and four 4 mm round scleral punches were made per eye from standardized locations around the optic nerve. Specifically, one punch was taken from each of the nasal, temporal, superior, and inferior equatorial regions. To ensure consistency across samples, punches were collected at equal distances from the optic nerve head using anatomical landmarks and orientation during dissection. Previous baseline experiments showed no detectable differences in thermal degradation temperature among these four equatorial regions. NIR illumination of the scleral punches was performed immediately. For transpupillary illumination, the whole WST-D–treated eyes were directly exposed to NIR light through the pupil, targeting the nasal equatorial region. After 30 min, the eyes were dissected, and a single 4 mm scleral punch was collected per eye, specifically from the nasal equatorial area that had been exposed to NIR light. The efficacy of the treatments was immediately measured by testing the treated scleral punches on a temperature degradation assay.

### WST11 retention assessment

WST11 was administered to the temporal equatorial sclera *in vivo*. To ensure continuous drug exposure, a 5 mm × 5 mm sponge (Spongostan Dental, Healthware Australia, Narellan, Australia) soaked in WST11 was placed in a conjunctival pocket over the sclera and left for 30 min, 5–24 h. The sponge remained securely positioned throughout the incubation period. Saline/NIR controls were included for the 30 min and 24 h time point. After each incubation period, the animals were euthanized, and a single 4 mm scleral punch was immediately collected from the treated temporal equatorial region of each eye. The punch was immediately subjected to NIR illumination, and the efficacy of the treatments was then directly measured using a thermal degradation assay, all performed consecutively on the same day at room temperature.

For the 30-minute time point, two animals were treated with WST-D/NIR in the right eye and received saline-D/NIR treatment in the left eye (Table 1). A third animal received WST-D/NIR treatment in both eyes. For the 24-hour time point, the right eye received WST-D/NIR treatment, and the left eye received saline-D/NIR treatment. At the 5-hour time point, the right eye was treated with WST-D/NIR, while the left eye served as an untreated control.

To distinguish between passive diffusion and active clearance, an additional 24-h post-mortem experiment was performed in one animal, using the right eye. WST11 was applied *in vivo* using the sponge, and after 30 min, the animals were euthanized. The eyes remained in situ for 24 h before dissection. Equatorial scleral punches were collected and immediately exposed to NIR illumination. The efficacy of the treatments was measured using the thermal degradation assay.

### Theoretical estimation of NIR penetration

To estimate the feasibility of NIR penetration through the sclera, we performed a theoretical analysis based on the Beer–Lambert law:

$$\:I\left(z\right)=I\left(0\right)\times\:{e}^{-\mu\:z}$$

where I(z) is the light intensity at depth z, I(0) is the incident light intensity at the tissue surface, µ is the effective attenuation coefficient (cm⁻¹), and z is the depth in tissue (cm). An attenuation coefficient relevant to 750 nm in biological tissue was applied. This simplified model assumes uniform, diffuse tissue scattering and absorption. The analysis was used to estimate the percentage of light reaching the outer scleral boundary at various depths.

### *In vivo* WST-D/NIR treatment

After inducing general anesthesia, a corneal suture (Suture 7 − 0, 18” Chromic Gut Monofilament TG140-8) was threaded adjacent to the limbus and the monofilament was weighted to gently rotate the eye to one side, exposing the sclera. To treat the sclera, WST-D was either added by a sub-Tenon injection (100 µl) to the temporal equatorial sclera, or a retrobulbar injection (300 µl) to the temporal posterior sclera. After 30 min, the WST-D–treated temporal equatorial or posterior sclera was illuminated by NIR light through the pupil. For animals treated at the equatorial region (N = 6), the left eye received saline-D/NIR treatment, and the right eye received WST-D/NIR treatment. For animals treated at the posterior region (N = 6), one eye per animal received WST-D/NIR treatment. After 30 min of illumination, the guinea pigs were euthanized, and the eyes were enucleated. From each eye, two 4 mm round scleral punches were collected: one from the treated temporal region that had been exposed to NIR light (the treated area), and one from the untreated nasal equatorial region, which served as the within-eye contralateral control. As an additional reference group, untreated scleral punches (N = 8) were collected from untreated eyes from separate animals. The efficacy of treatment was immediately assessed using the thermal degradation assay.

### Thermal degradation assay

A thermal degradation assay is a technique used to study collagen crosslinking. Collagen fibers progressively shrink when heated, with structural disruption occurring once hydrogen bonds in the triple helix break, leading to helix unwinding^61^. The denaturation of the tertiary protein structure leads to rapid tissue shrinkage. This shrinkage can be quantified by key temperature points, including the temperature at maximal shrinkage rate^68^, with collagen fiber stabilization through crosslinking resulting in an increase in temperature.

Differential scanning calorimetry, a widely used method for assessing biomaterial crosslinking, is closely related to a thermal degradation assay, with thermal shrinkage temperature serving as a simplified yet effective alternative for evaluating crosslinking efficacy^69^. The increase in thermal stability correlates with enzymatic resistance, as aldehyde-induced crosslinking stabilizes collagen against both thermal degradation and enzymatic breakdown by collagenase. The order of thermal stability conferred by aldehydes (formaldehyde > glutaraldehyde > glyoxal > crotonaldehyde) mirrors their effect on enzymatic resistance, indicating that the number of cross-links formed influences both properties in a similar manner^30^. This further supports the use of thermal degradation assays as a reliable method to assess crosslinking efficacy. To assess treatment efficacy, we determined the maximal rate of change in tissue shrinkage or more simply, the temperature required for 50% tissue shrinkage (*T*_*50*_). Hereby, *ΔT*_*50*_ is the change in *T*_*50*_ as compared to untreated control. We first used the thermal degradation assay to efficiently screen and optimize WST-D/NIR treatment parameters, as it enables localized and higher-throughput evaluation of collagen stabilization. We then performed tensile testing on *ex vivo* scleral tissue to calculate the Young’s modulus, using the optimized parameters to directly verify treatment-induced changes in stiffness and to confirm that the thermal assay reflects underlying biomechanical alterations. In the* in vivo* experiments, where the transpupillary treatment produced a small posterior scleral spot that only permitted punch biopsies, strip-based mechanical testing was not feasible; thus, the thermal degradation assay was used to assess collagen stabilization in the localized treated region. To assess the efficacy of the WST-D/NIR treatment using a thermal degradation assay, scleral punches were mounted into the wells of a solid copper block containing phosphate-buffered saline (PBS) (Supplementary Fig. 9). Punches were prevented from floating using a custom-made fine grid placed on top of the punches, after which the wells were coverslipped to prevent evaporation. The copper block was then heated at 1 °C/min while being imaged with an overhead camera at every 1 °C increment between 55 °C and 70 °C. The surface area of each scleral punch was measured at each temperature point using ImageJ (version 1.54d, National Institutes of Health, Bethesda, USA). For each image, the scleral punch boundary was manually outlined to determine surface area. The area was normalized relative to the initial scleral area at 55 °C. The percentage change was calculated as a function of temperature. A curve was fitted to the data for each sample using a sigmoid 5 parameter fit to determine the temperature at 50% tissue degradation (*T*_*50*_).

### Tensile testing

Circumferential strips were cut from the sclera to approximately 3.5 mm in width and 10 mm in length using surgical scissors on a soft base. Specimens were maintained in PBS during preparation and throughout testing to prevent dehydration. Specimen thickness was measured prior to mechanical testing using a non-contact laser displacement sensor (CL-P030, KEYENCE, Osaka, Japan). Specimens were mounted between mechanical clamps with an initial gauge length of 4 mm. Uniaxial tensile tests were performed on a TA ElectroForce 3200 mechanical tester (TA Instruments, New Castle, USA) equipped with a 9.8 N load cell (TA Instruments). Each specimen was first preconditioned with five loading–unloading cycles to 0.1 N at an extension rate of 1 mm/min, after which a 10 s pause was applied before the specimen was subjected to tensile loading at 1 mm/min until failure. Force–displacement data were recorded and converted into stress–strain curves, where stress was calculated as the applied load divided by the mean cross-sectional area (width × thickness), and strain as the elongation divided by the initial specimen length. From the stress–strain curves, the Young’s modulus was derived, representing the stiffness of the tissue. Specimens were kept hydrated with PBS throughout the tests.

### Statistical analysis

Data were tested for normality using the Shapiro-Wilk test, with all groups passing normality (*p* > 0.05), and reported as the mean ± standard error (SE). One-way ANOVA was used to compare the *ex vivo* and* in vivo* efficacy across different treatment conditions, to each other and to the untreated control group. The Brown-Forsythe test was applied to assess homogeneity of variances, and since variances were found to be unequal (*p* < 0.0001), Welch’s ANOVA was used to account for the variance differences. Post-hoc analysis was conducted using the Dunnett T3 test to compare each treated group to their controls and performs pairwise comparisons between treated groups, accounting for unequal variances. All statistical analyses were performed using GraphPad Prism (version 10.3.1, San Diego, USA) with significance set at *p* < 0.05.

## Supplementary Information

Below is the link to the electronic supplementary material.

Supplementary Material 1

## Data Availability

The datasets generated during and analyzed during the current study are available from the corresponding author on reasonable request.
