# Supplementary material for: A transpupillary approach for crosslinking Guinea pig sclera using WST11 and near-infrared light
Source: Sci Rep. 2026 Jan 24;16:6098. doi: 10.1038/s41598-026-36438-w (PMC12902051; doi:10.1038/s41598-026-36438-w)
Supplement: Supplementary file 1 — Supplementary Material 1 [file 41598_2026_36438_MOESM1_ESM.docx]

**Supplementary figures**

**Supplementary Figure 1.**


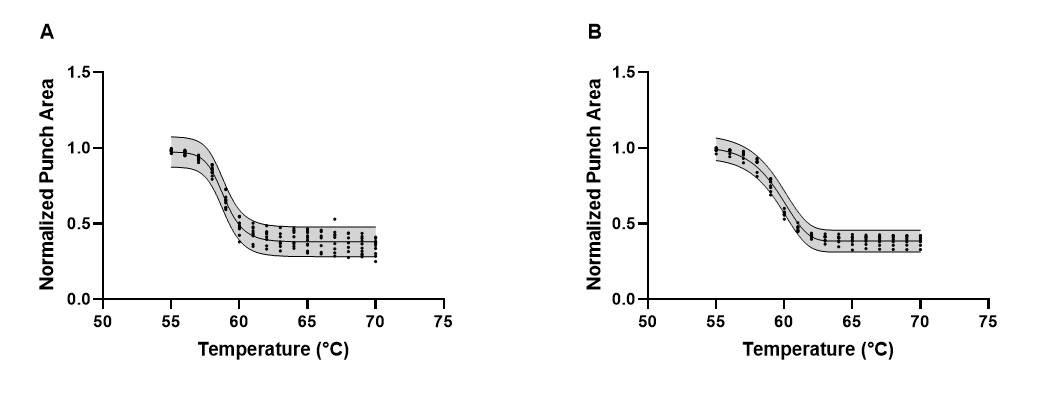


Supplementary Figure 1. Thermal degradation assay of untreated sclera from 1- to 2-month-old (A) and 5- to 6-month-old (B) guinea pigs. The normalized punch area, calculated as the scleral surface area relative to its initial area, is plotted as a function of temperature (°C). A sigmoidal 5-parameter curve was fitted to the data for each condition. The gray area represents the 95% prediction bands, which enclose the region expected to contain 95% of future data points. These bands account for both the uncertainty in the true position of the curve and the scatter of data around the fitted curve.

**Supplementary Figure 2.**


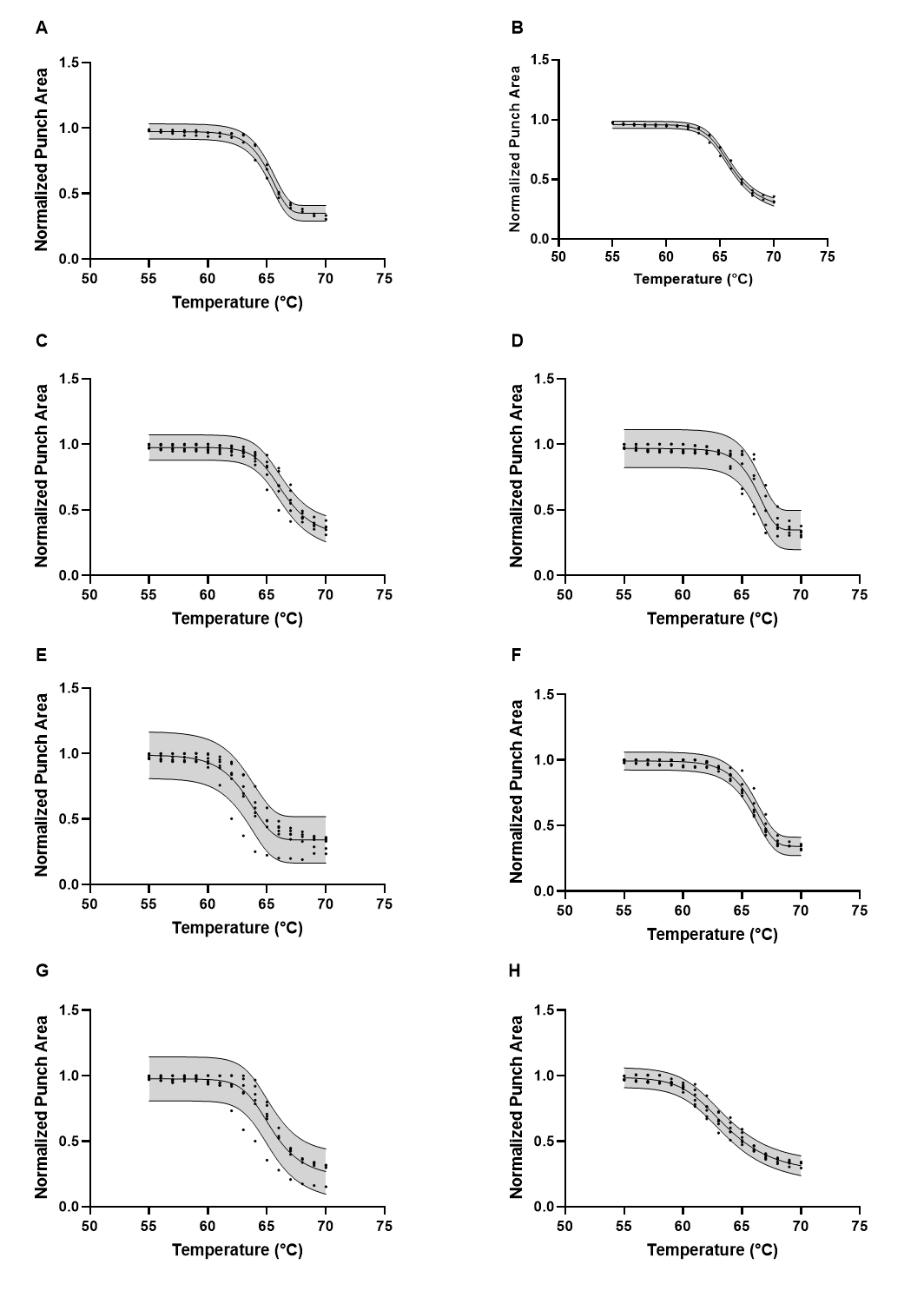


Supplementary Figure 2. *Ex vivo* optimization of laser time and power for protection against thermal degradation, measured by a thermal degradation assay. Guinea pig sclera from 5- to 6-month-old guinea pigs were treated with WST11 + 10% dextran for 30 min followed by various laser irradiation times and powers: (A) 10 min at 10 mW/cm^2^, (B) 20 min at 10 mW/cm^2^, (C) 30 min at 10 mW/cm^2^, (D) 60 min at 10 mW/cm^2^, (E) 10 min at 20 mW/cm^2^, (F) 20 min at 20 mW/cm^2^, (G) 30 min at 20 mW/cm^2^. (H) For comparison, sclera from younger 1- to 2-month-old guinea pigs were treated with WST + 10% dextran for 30 min followed by NIR illumination for 30 min at 10 mW/cm^2^. The normalized punch area, calculated as the scleral surface area relative to its initial area, is plotted as a function of temperature (°C). A sigmoidal 5-parameter curve was fitted to the data for each condition. The gray area represents the 95% prediction bands, which enclose the region expected to contain 95% of future data points. These bands account for both the uncertainty in the true position of the curve and the scatter of data around the fitted curve.


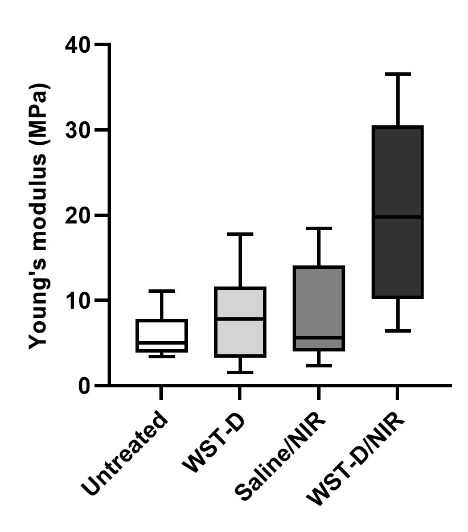
**Supplementary Figure 3.**

Supplementary Figure 3. *Ex vivo* biomechanical testing of guinea pig sclera following WST-D/NIR treatment. Box plots showing Young’s modulus values for untreated control, saline + NIR, WST11 + 10% dextran (WST-D) alone, and WST-D/NIR (WST11 + 10% dextran followed by NIR illumination) groups (N = 8 for each group). For the NIR conditions, illumination was performed for 30 minutes at 10 mW/cm². Pairwise comparisons showed no significant differences between untreated, saline/NIR, and WST-D groups (*p* > 0.05), whereas the WST-D/NIR group exhibited significantly higher stiffness compared to untreated (*p* = 0.0014), WST-D (*p* = 0.0074), and saline/NIR (*p* = 0.0067). Boxes represent interquartile ranges, horizontal lines indicate medians, whiskers show range, and dots denote individual measurements.

**Supplementary Figure 4.**


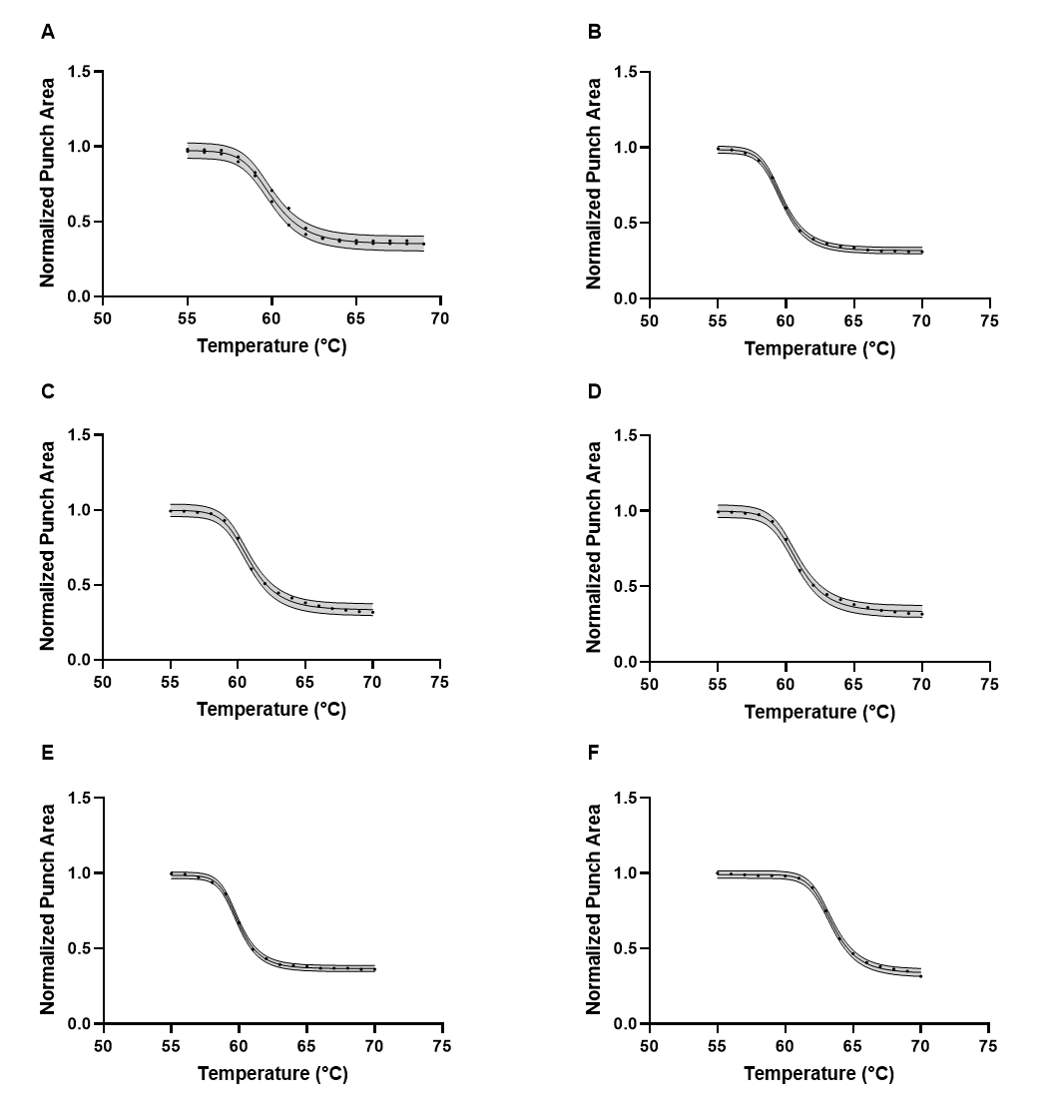


Supplementary Figure 4. Assessment of the retention of WST11 in the sclera over time, measured by a thermal degradation assay. Saline (A, B) or WST11 (C–E) was administered to the equatorial sclera *in vivo* and incubated for 30 min (A, C; N = 2 and 4, respectively), 5 h (D; N = 1) or 24 h (B, E; N = 1 each). After each incubation period, equatorial scleral punches were immediately illuminated with NIR light at a power of 10 mW/cm^2^ for 30 min. (F) To distinguish between passive diffusion and active clearance, we delayed NIR illumination for 24 h post-mortem. Guinea pig sclera was incubated with WST11 for 30 min, the animal was euthanized, and the eyes remained *in situ* for 24 h before dissection, and equatorial scleral punches exposed to NIR illumination at 10 mW/cm² for 30 min. The efficacy of the treatments was measured using the thermal degradation assay. The normalized punch area, calculated as the scleral surface area relative to its initial area, is plotted as a function of temperature (°C). A sigmoidal 5-parameter curve was fitted to the data for each condition. The gray area represents the 95% prediction bands, which enclose the region expected to contain 95% of future data points. These bands account for both the uncertainty in the true position of the curve and the scatter of data around the fitted curve.

**Supplementary Figure 5.**


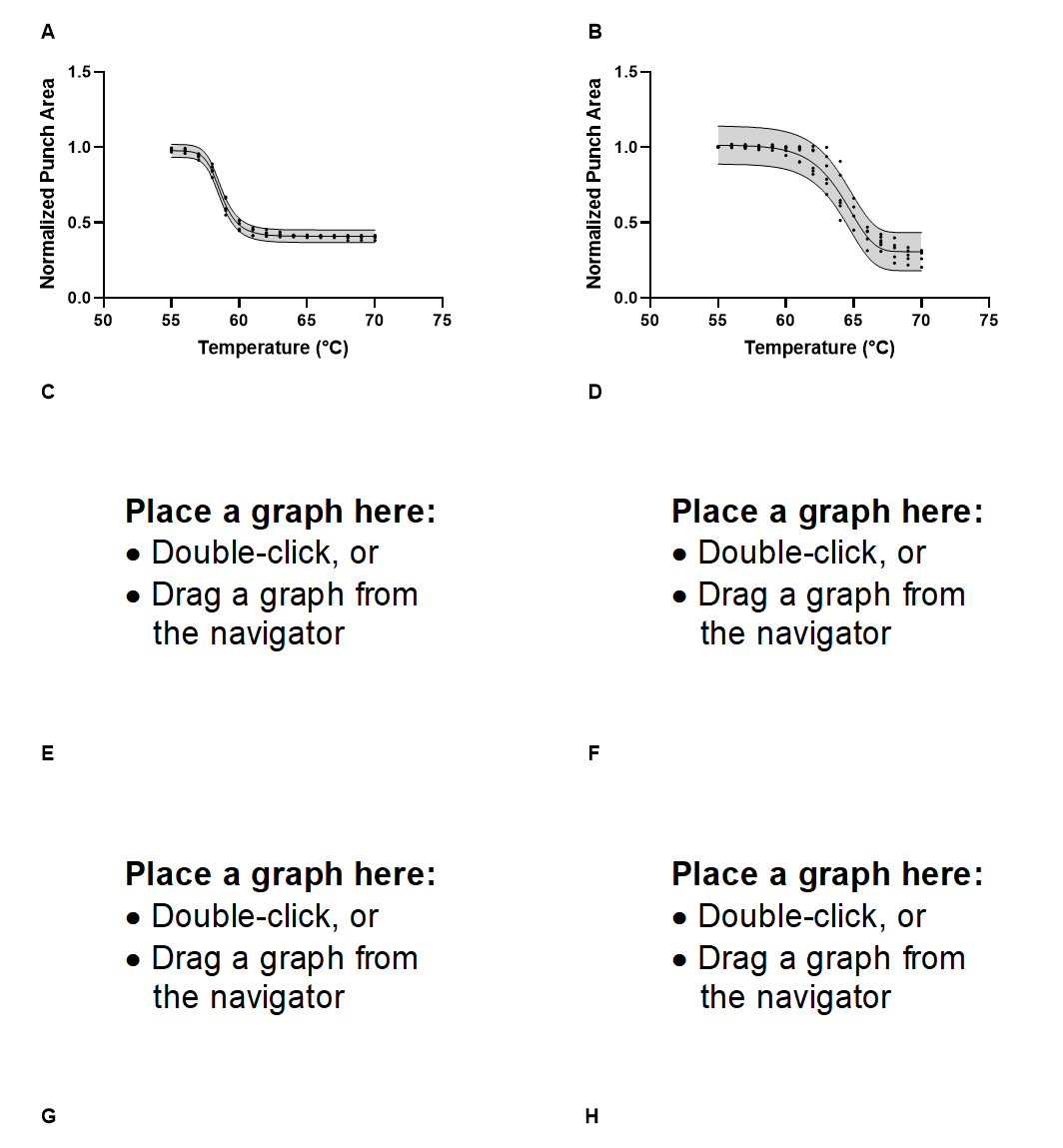


Supplementary Figure 5. Validation of transpupillary illumination *ex vivo* using whole enucleated eyes from 1- to 2-month-old guinea pigs. Guinea pig eyes were incubated for 30 min in either saline (A) or WST11 + 10% dextran (B), then followed by transpupillary NIR illumination at 10 mW/cm2 for 30 min, targeting the equatorial sclera. The efficacy of the treatments was measured using a thermal degradation assay. The normalized punch area, calculated as the scleral surface area relative to its initial area, is plotted as a function of temperature (°C). A sigmoidal 5-parameter curve was fitted to the data for each condition. The gray area represents the 95% prediction bands, which enclose the region expected to contain 95% of future data points. These bands account for both the uncertainty in the true position of the curve and the scatter of data around the fitted curve.

**Supplementary Figure 6.**


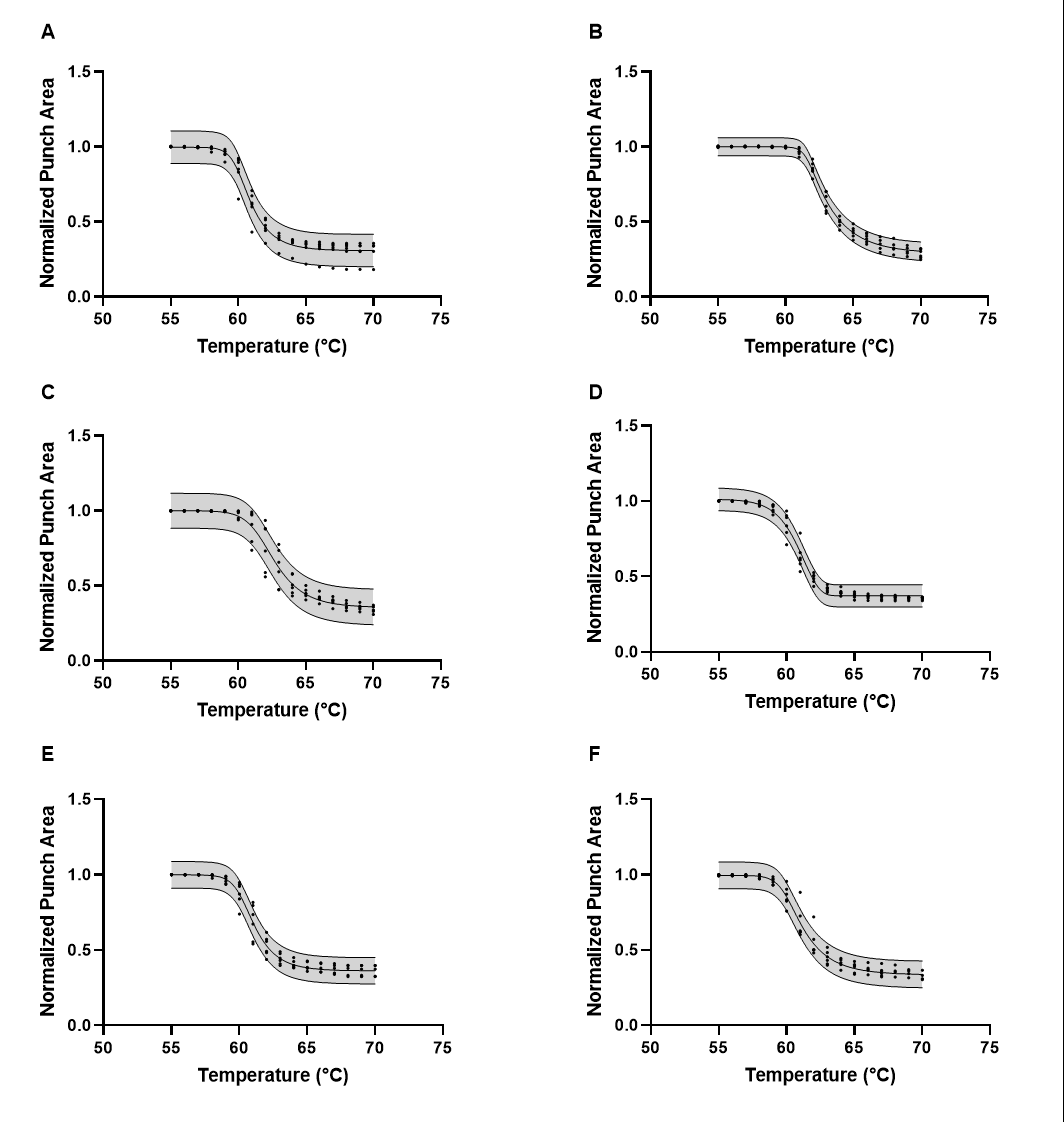


Supplementary Figure 6. *In vivo* efficacy of WST-D/NIR treatment in 6-month-old guinea pigs. (A) Saline +10% dextran treatment via a sub-Tenon injection delivered to the equatorial sclera. (B) Equatorial WST11 +10% dextran treatment via a sub-Tenon injection. (C) Posterior WST11 +10% dextran treatment via a sub-retractor bulbi muscle injection. Thirty minutes after each injection, NIR transpupillary irradiation at 15 mW/cm^2^ for 30 min was delivered to the sclera. To assess the localized or potential general effect beyond the treatment area, the contralateral side of the equatorial saline-D/NIR control (D), equatorial WST-D/NIR treatment (E) and posterior WST-D/NIR treatment (F) were also analyzed. The efficacy of each treatment was measured using a thermal degradation assay. The normalized punch area, calculated as the scleral surface area relative to its initial area, is plotted as a function of temperature (°C). A sigmoidal 5-parameter curve was fitted to the data for each condition. The gray area represents the 95% prediction bands, which enclose the region expected to contain 95% of future data points. These bands account for both the uncertainty in the true position of the curve and the scatter of data around the fitted curve.

**Supplementary Figure 7.**


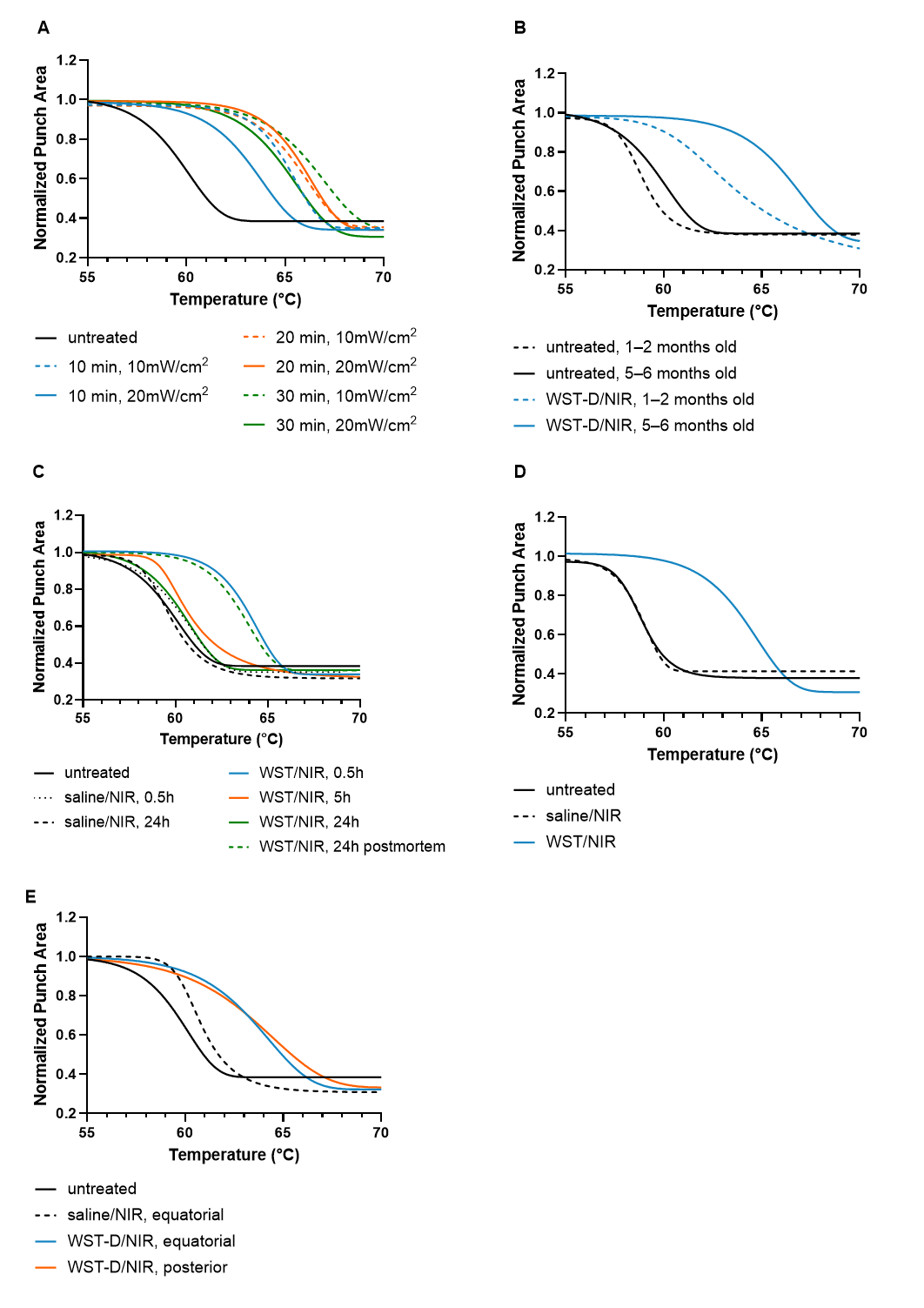


Supplementary Figure 7. Comparison of fitted sigmoidal curves for the efficacy of various WST-D/NIR treatments. Sigmoidal 5-parameter curves were fitted to the data from the thermal degradation assay for each treatment condition. The curves represent the normalized punch area, calculated as the scleral surface area relative to its initial area, plotted against temperature (°C) for each condition per experiment: (A) optimization of NIR irradiation time and power; (B) effect of age on the efficacy of the treatment; (C) time-dependent retention of WST11; (D) validation of *ex vivo* transpupillary NIR illumination; (E) *in vivo* efficacy of WST-D/NIR treatment.


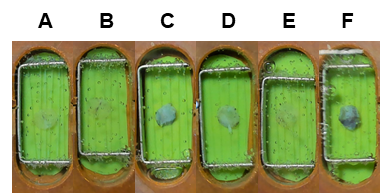
**Supplementary Figure 8**

Supplementary Figure 8. Representative images of guinea pig sclera during the WST11 retention assessment using a thermal degradation assay at 70 °C. Saline or WST11 was administered to the equatorial sclera *in vivo* and incubated for 30 min, 5 h, or 24 h before dissection; untreated sclera served as control. After each incubation period, equatorial scleral punches were illuminated with NIR light at 10 mW/cm² for 30 min. (A) Untreated control, (B) saline/NIR control, (C) WST-D/NIR after 30 min incubation with immediate illumination, (D) WST-D/NIR after 5 h incubation, (E) WST-D/NIR after 24 h incubation, and (F) WST-D/NIR after 30 min incubation followed by 24 h in situ post-mortem before NIR illumination. Sclerae in the 30 min and 24 h post-mortem groups (C, F) appeared opaque, reflecting the presence of WST11; the 5 h group (D) was slightly opaque, while the 24 h in vivo group (E) appeared fully translucent, similar to untreated and saline controls (A, B), indicating clearance of WST11.

**
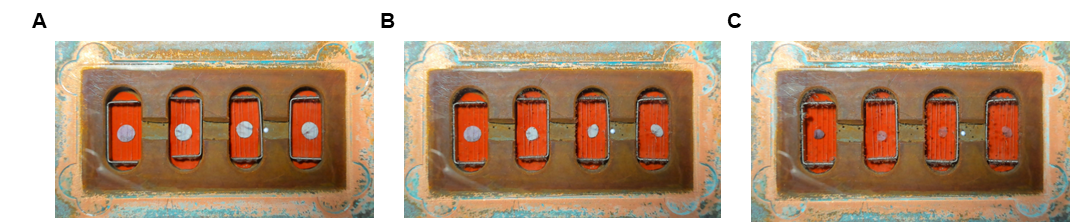
Supplementary Figure 9.**

Supplementary Figure 9. Representative images of scleral punches during the thermal degradation assay, captured at different temperature points on the copper heating block. The scleral punches shown were obtained from a single 6-month-old guinea pig in the *in vivo* equatorial experiment, in which the right eye was treated with WST-D/NIR and the left eye with saline-D/NIR. From each eye, two 4 mm round scleral punches were collected: one from the treated temporal region that had been exposed to NIR light, and one from the untreated nasal equatorial region, which served as the contralateral control. Each image (A–C) shows four wells, each containing one scleral punch. From left to right: well 1 contains WST-D/NIR-treated sclera; well 2, the contralateral control of the WST-D/NIR-treated eye; well 3, saline-D/NIR-treated sclera; and well 4, the contralateral control of the saline-D/NIR-treated eye. Images are shown for three representative temperatures: (A) 55 °C, (B) 62 °C, and (C) 70 °C. Surface area measurements were performed at every 1 °C increment between 55 °C and 70 °C using similar images acquired throughout the heating process.
